# Beneficial effects of fingolimod in MS patients with high serum Sema4A levels

**DOI:** 10.1371/journal.pone.0193986

**Published:** 2018-03-08

**Authors:** Toru Koda, Akiko Namba, Yuji Nakatsuji, Masaaki Niino, Yusei Miyazaki, Tomoyuki Sugimoto, Makoto Kinoshita, Kazushiro Takata, Kazuya Yamashita, Mikito Shimizu, Toshiyuki Fukazawa, Atsushi Kumanogoh, Hideki Mochizuki, Tatsusada Okuno

**Affiliations:** 1 Department of Neurology, Osaka University Graduate School of Medicine, Suita, Osaka, Japan; 2 Department of Neurology, Toyama University Hospital, Toyama, Toyama, Japan; 3 Department of Clinical Research, Hokkaido Medical Center, Sapporo, Hokkaido, Japan; 4 Graduate School of Science and Engineering, Kagoshima University, Kagoshima, Kagoshima, Japan; 5 Department of Neurology, Osaka General Medical Center, Osaka, Osaka, Japan; 6 Sapporo Neurology Hospital, Sapporo, Hokkaido, Japan; 7 Department of Respiratory Medicine, Allergy and Rheumatic Diseases, Osaka University Graduate School of Medicine, Suita, Osaka, Japan; Nagoya Daigaku, JAPAN

## Abstract

We previously demonstrated that patients with multiple sclerosis (MS) of high serum Sema4A levels are resistant to IFN-β therapy. To further elucidate the role of serum Sema4A as a biomarker for therapeutic stratification in MS patients, it is important to clarify the efficacy of other disease-modifying drugs (DMD) in those with high serum Sema4A levels. Thus, in this study we investigated whether fingolimod has beneficial effects on MS patients with high Sema4A levels. We retrospectively analyzed annualized relapse rate (ARR) and Expanded Disability Status Scale (EDSS) change in 56 relapsing—remitting multiple sclerosis (RRMS) patients who had been treated with fingolimod, including those who switched from IFN-β therapy. The levels of Sema4A in the sera were measured by sandwich ELISA. The implications of Sema4A on the efficacy of fingolimod were investigated by administering recombinant Sema4A-Fc and fingolimod to mice with experimental autoimmune encephalomyelitis (EAE). Retrospective analysis of MS cohort (17 high Sema4A and 39 low Sema4A) demonstrated the effectiveness of fingolimod in those with high serum Sema4A levels, showing reduction of ARR (from 1.21 to 0.12) and EDSS progression (from 0.50 to 0.04). Consistent with this observation, improvement in the disease severity of EAE mice receiving recombinant Sema4A-Fc was also observed after fingolimod treatment. These data suggest that fingolimod could serve as a candidate DMD for managing the disease activity of MS patients with high Sema4A levels.

## Introduction

Multiple Sclerosis (MS) is an inflammatory demyelinating disease of the central nervous system (CNS) [1]. In addition to IFN-β and glatiramer acetate (GA), a variety of disease-modifying drugs (DMDs) including fingolimod have been recently developed and applied in MS treatment [2]. Although one clinical trial suggested the superiority of fingolimod to IFN-β in its efficacy [3], cardiovascular adverse events, infections, macular edema, and progressive multifocal leukoencephalopathy (PML) have raised concerns during the treatment with fingolimod [4]. Indeed, US Food and Drug Administration (FDA) approved fingolimod as a first-line DMD, while European Medicines Agency (EMA) licensed fingolimod as a second-line agent due to its safety issues.

Sema4A, a membrane-type class IV semaphorin, plays an important role in the activation and differentiation of Th cells [5]. We previously demonstrated that Sema4A is increased in the sera of patients with MS, and those with high serum Sema4A levels do not respond to IFN-β therapy [6]. Consistent with these clinical observations, we showed IFN-β non-responsiveness in experimental autoimmune encephalomyelitis (EAE) mice with high serum Sema4A state [7]. Thus, it is important to address whether fingolimod has beneficial effects in patients with high serum Sema4A levels, representing non-responders to first-line IFN-β therapy.

In this study, we retrospectively analyzed whether patients with high serum Sema4A levels show favorable response to fingolimod therapy, and investigated the efficacy of fingolimod in an EAE model with high serum Sema4A state.

## Materials and methods

### Subjects and measurement of serum Sema4A

All 56 relapsing—remitting multiple sclerosis (RRMS) patients who had been treated with fingolimod in the following institutions were enrolled at Osaka University Hospital (n = 16), Hokkaido Medical Center (n = 25), and Sapporo Neurology Clinic (n = 15) and retrospectively analyzed in Osaka University Graduate School of Medicine. All subjects fulfilled the 2010 McDonald criteria, and 48 patients had been treated with DMDs before fingolimod switching (IFN-β, n = 46 (13; Osaka University Hospital, 11; Hokkaido Medical Center, 22; Sapporo Medical Center), Dimethyl Fumarate, n = 1 (Hokkaido Medical Center), Natalizumab, n = 1 (Osaka University Hospital).

Patients with neuromyelitis optica spectrum disorders (NMOSD) were excluded from the cohort. Blood samples were collected more than 3 months before or after fingolimod treatment. The levels of Sema4A in the sera were analyzed by sandwich ELISA as previously described [6]. Sample analysis was conducted blindly without clinical information.

### Clinical assessment

The number of relapses and Expanded Disability Status Scale (EDSS) scores were analyzed. EDSS scores were obtained via certified raters. Relapse was defined as the appearance of new symptoms or signs that lasted more than 24 hours without concurrent fever or illness. Relapses of patients were examined by experienced neurologists.

### Animals & reagents

Sema4A-Fc protein was prepared as previously described [5]. Briefly, we generated recombinant soluble mouse Sema4A protein comprising the putative extracellular region fused to the human immunoglobulin 1 (IgG1) Fc fragment. For IFN-β treatment in EAE, IFN-β 1b (PBL Biomedical Laboratories, Piscataway, NJ) or phosphate buffered saline (PBS) was administered to immunized mice.

### Induction of EAE and treatments

Wild-type C57BL/6 female mice were purchased from Oriental Yeast Corp. (Tokyo, Japan) and were maintained in a specific pathogen-free environment. All possible efforts were made to minimize animal suffering and limit the number of animals used. EAE was induced using a modification of our previously reported method [8, 9]. In brief, 8 to 10 weeks old C57BL/6 female mice were subcutaneously injected with 100 μg myelin oligodendrocyte glycoprotein (MOG) _35–55_ emulsified in complete Freund's adjuvant (CFA) supplemented with intraperitoneal injections of 400 ng pertussis toxin (List Laboratories, Campbell, CA, USA) on days 0 and 2.

Fingolimod (0.03 mg/kg body weight) or saline was given by oral gavage or intraperitoneal injection once daily after immunization. In addition, Sema4A-Fc (20 μg) or IFN-β 1b (10,000 U) or control human IgG (20 μg) was intravenously injected on days 0, 1, 3, 5, 7, 9 and 10 after immunization.

All mice were monitored daily for clinical signs and were scored as follows using a scale of 0–5: 0, no overt signs of disease; 1, limp tail; 2, hind limb paralysis; 3, complete hind limb paralysis; 4, complete forelimb paralysis; 5, moribund state or death. Blood samples were collected 22 days after immunization. The peripheral blood lymphocyte (PBL) counts were obtained for each blood sample using Vetscan HM2 (ABAXIS, Wemmel, Belgium).

### Statistical analysis

One-way analysis of student T-tests was used to compare two groups. EAE data are presented as means ± SEM. Statistical significance was determined with one-way ANOVA. p-values less than or equal to 0.05 were considered statistically significant.

### Standard protocol approvals, registrations, and patient consents

All subjects gave informed consent prior to participation. This study was approved by the ethical committee of Osaka University Hospital (Permit Number: 12091–6). Experimental procedures were approved by the Animal Care and Use Committee of Osaka University Graduate School of Medicine (Permit Number: 20-084-6). Mice were housed in microisolator cages at a modified pathogen-free barrier facility in the Animal Resource Center for Infectious Diseases, Research Institute for Microbial Diseases, Osaka University. All of the experimental procedures were performed following our institutional guidelines. Mice had free access to food and water ad libitum, and sodium pentobarbital anesthesia was applied in all of the surgery performed. All necessary steps were taken to ameliorate suffering to animals involved in our study, and mice were euthanized by CO_2_ inhalation. None of the mice in our study reached the criteria of defined humane endpoints. Mortality outside our planned euthanasia or humane endpoints neither occurred.

## Results

### Fingolimod reduces ARR among patients with high serum Sema4A levels

**Fig 1:**
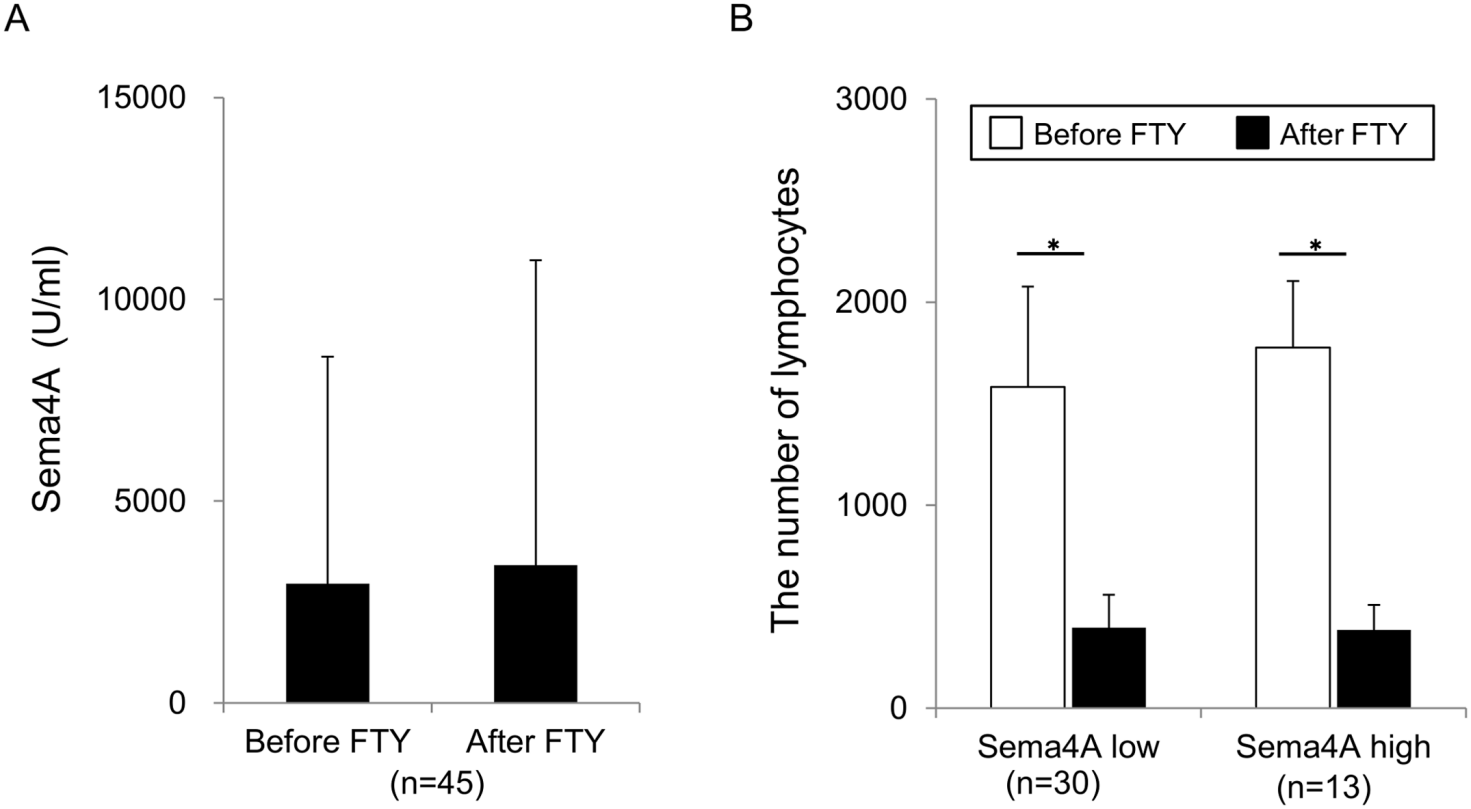
Effect of fingolimod on the levels of Sema4A and number of lymphocytes. (A) The levels of Sema4A are not affected by fingolimod. The serum Sema4A levels were measured before and 3 months after fingolimod therapy. Fingolimod therapy did not affect the Sema4A levels (p = 0.675, Welch’s t test). (B) The Sema4A levels did not affect lymphopenia under fingolimod treatment. The cell counts of lymphocytes were measured before and 3 months after fingolimod therapy. Fingolimod-induced lymphopenia were observed in both patients with high Sema4A levels and those with low Sema4A levels. Data represent the mean ± SD. *p ≤ 0.05.

**Fig 2:**
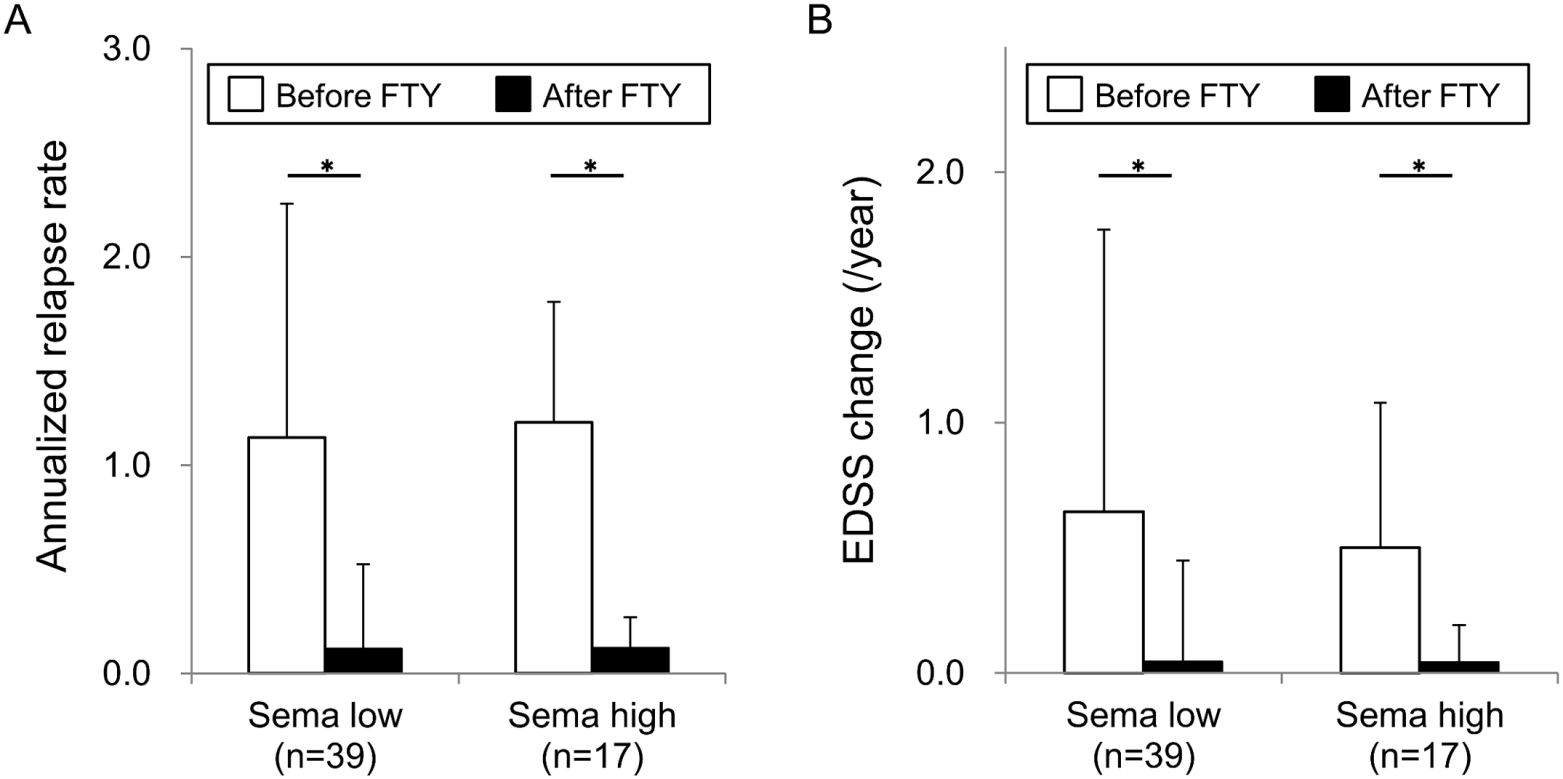
Fingolimod is effective for patients with high Sema4A. (A) Annualized relapse rate (ARR) before and after fingolimod treatment is shown. Fingolimod reduced ARR both for patients with low Sema4A levels and for those with high Sema4A levels. Data represent the mean ± SD. *p ≤ 0.05. (B) EDSS progression (EDSS annual change) after fingolimod treatment is shown. There was no difference between patients with low Sema4A levels and those with high Sema4A levels.

To investigate the clinical relevance of the distinct modes of action of fingolimod and IFN-β therapies in MS patients with various Sema4A levels, we first examined the correlation of serum Sema4A levels and fingolimod efficacy among the patients recruited in our study. Demographic features of fingolimod cohort are shown in Table 1. The average serum Sema4A titers of the cohort were 2877 ± 5469 U/ml, with patients of high levels of serum Sema4A (>2500 U/ml) accounting for 30% of all cases. Other clinical parameters, including the percentage of female per male, age at examination, age at onset, disease duration, duration from onset to fingolimod treatment, durations of fingolimod treatment, relapse number per 2 years before fingolimod treatment, EDSS at induction of fingolimod treatment, peripheral blood lymphocyte numbers before fingolimod treatment, positive ratio of oligoclonal IgG band were not significantly different between high and low serum Sema4A groups. Since the levels of serum Sema4A were not changed by IFN-β treatment or relapses in our previous study, we first examined whether the levels of Sema4A will be affected by fingolimod treatment or not. The serum levels of Sema4A did not alter by fingolimod treatment (Fig 1A). PBL counts were decreased in patients with both high and low Sema4A levels (Fig 1B). With regard to the efficacy of fingolimod in patients with high and low Sema4A levels, fingolimod reduced the ARR (Fig 2A) and EDSS progression per year (Fig 2B) in patients with both high and low serum Sema4A levels. Thus, serum Sema4A levels were irrelevant to the pharmacological effect of fingolimod on PBL counts, and more importantly did not affect the efficacy of fingolimod in inhibiting relapses and progression of MS.

**Table 1:** Clinical characteristics of subjects in fingolimod cohort.

	Total (56)	Sema4A high (17)	Sema4A low (39)	p value
Female/Male (% female)	36/20 (64.3)	11/6 (64.7)	25/14 (64.1)	1.000
Age at examination (mean ± SD, years)	41.8 ± 10.5	39.9 ± 12.7	42.7 ± 9.4	0.377
Age at onset (mean ± SD, years)	28.6 ± 8.6	26.6 ±8.1	29.4 ± 8.8	0.268
Disease duration (mean ± SD, years)	13.3 ± 9.6	13.3 ± 10.9	13.2 ± 9.1	0.970
Duration from onset to FTY treatment (mean ± SD, years)	11.2 ± 9.6	11.1 ± 11.0	11.2 ± 9.1	0.956
Duration of FTY treatment (mean ± SD, years)	1.96 ± 0.9	2.19 ± 0.7	1.85 ± 1.0	0.217
Sema4A (mean ± SD, U/mL)	2877 ± 5469	9233 ± 7456	517 ± 737	0.001
Relapse number/2 y before FTY (mean ± SD, /years)	2.31 ± 1.9	2.41 ± 2.0	2.26 ± 1.9	0.793
EDSS at induction of FTY (mean ± SD)	3.32 ± 2.3	3.09 ± 2.5	3.42 ± 2.5	0.624
The ratio of IFN treatment history (%)	82.1	82.4	82.1	1.000
PBL before FTY (mean ± SD, /mm3)	1644 ± 460	1776 ± 328	1582 ± 495	0.121
OB n/N (% positive)	26/35 (70.3)	9/10 (90.0)	17/27 (63.0)	0.233

### Fingolimod is effective in IFN-β non-responders with high Sema4A levels

**Fig 3:**
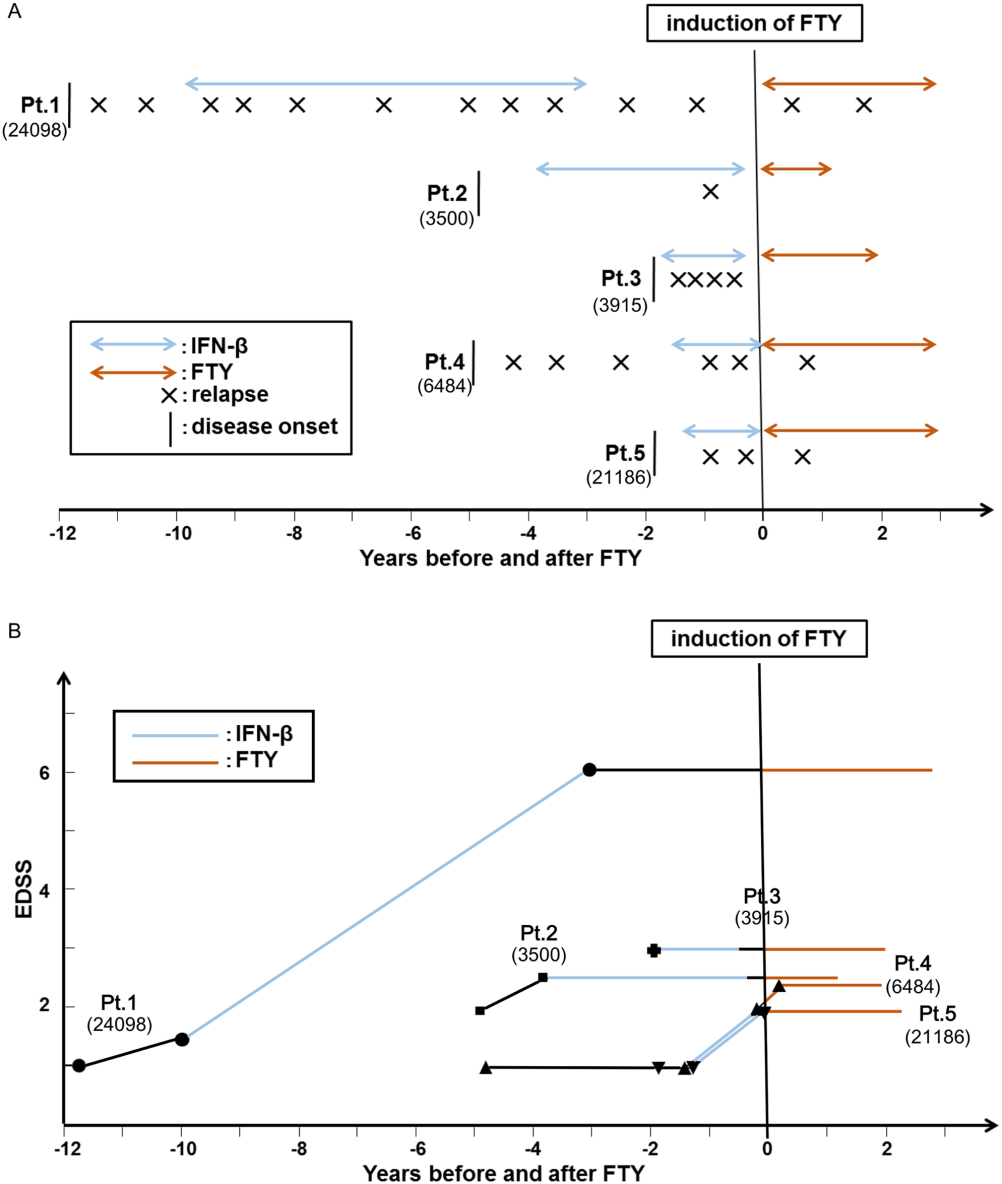
Fingolimod is effective in IFN-β non-responder patients with high Sema4A. Relapses (A) and EDSS (B) of all the patients with high Sema4A in Osaka University Hospital who changed from IFN-β to fingolimod treatment are shown. (The orange and blue lines represent duration of treatment with fingolimod and IFN-β, respectively.) During IFN-β treatment, the patients had many relapses and disease progressed. However, fingolimod tended to be effective in reducing the relapse rate (1.36 ± 0.80 (IFN-β) to 0.27 ± 0.25 (FTY), p = 0.054; Mann-Whitney U test) and in preventing further increase in EDSS in all patients (0.45 ± 0.37 (annual EDSS change, IFN-β) to 0.0 ± 0.0 (annual EDSS change, FTY), p = 0.046; Mann-Whitney U test). The Sema4A levels (U/ml) of each patient are as follows: Pt.1; 24098, Pt.2; 3500, Pt.3; 3915, Pt.4; 6484, Pt.5; 21186, indicated within the parenthesis.

Previously, we reported that patients with high Sema4A levels have more severe physical disabilities compared to those with low Sema4A levels under IFN-β treatment. This observation was confirmed in our present cohort, which is larger than that in the previous study (Data not shown). Next, we analyzed whether IFN-β non-responders with high Sema4A levels will benefit from fingolimod therapy. We retrospectively investigated chronological changes of ARR (Fig 3A) and EDSS score (Fig 3B) of all patients in Osaka University Hospital (n = 5) who had previous history of IFN-β injections and then switched to fingolimod therapy due to recurrent attacks within three years after the initiation of IFN-β therapy. Three of these patients had an increase of EDSS score within one year from the start of IFN-β treatment. Of note, EDSS score did not increase after fingolimod treatment in all patients. Additionally, patients 2 and 3 did not have relapses after switching to fingolimod treatment, while others experienced reduction in their relapse rate. These data suggest that fingolimod is effective in patients with high serum Sema4A levels who were unresponsive to IFN-β treatment.

### Therapeutic efficacy of fingolimod in EAE mice given Sema4A-Fc

**Fig 4:**
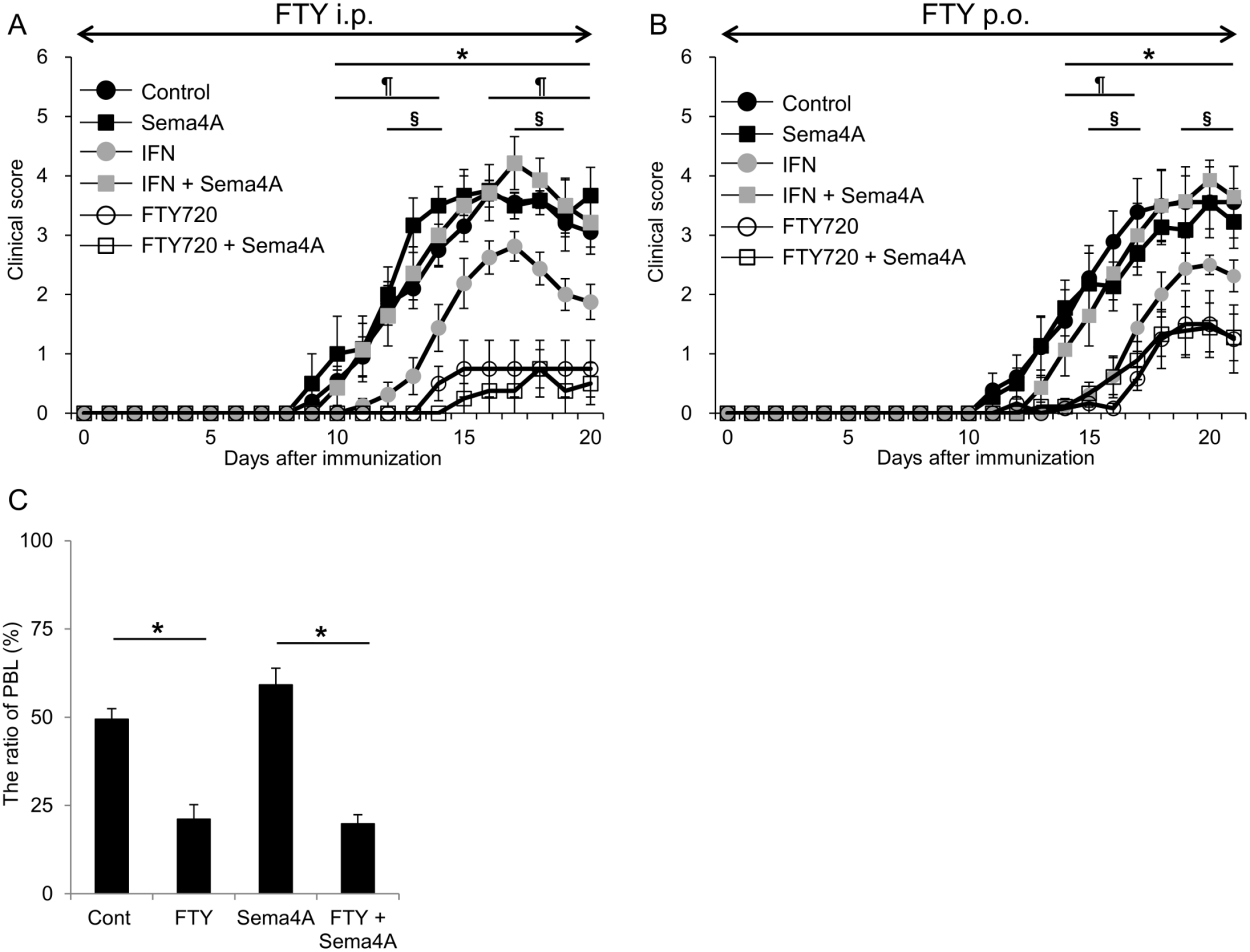
Fingolimod is effective for mice with EAE given Sema4A-Fc. (A), (B) The mean clinical scores of mice with EAE are shown. Immunized mice were treated with PBS + human IgG (Control; filled squares, n = 10), PBS + Sema4A-Fc (Sema4A; filled triangles, n = 8), IFN-β + human IgG (IFN; filled diamonds, n = 7 in (A), 8 in (B)), IFN-β + Sema4A-Fc (IFN + Sema4A; open circles, n = 7), Fingolimod + human IgG (FTY720; open squares, n = 8) or Fingolimod + Sema4A-Fc (FTY720 + Sema4A; open triangles, n = 8). Fingolimod is effective in mice with EAE given Sema4A-Fc. Administration method of fingolimod was intraperitoneal (i.p.) in (A) and oral (p.o.) in (B, C). Data represent the mean score ± SEM of two independent experiments. *p ≤ 0.05 for Fingolimod versus Control; ¶p ≤ 0.05 for IFN versus Control.; §p ≤ 0.05 for IFN+ Sema4A versus IFN. (C) The ratio of peripheral blood lymphocytes of mice with EAE at day 22 after immunization. *p ≤ 0.05.

MS patients with high serum Sema4A levels tend to be IFN-β non-responders. In accordance with this observation, we previously showed that recombinant Sema4A-Fc inhibited therapeutic effects of IFN-β in an animal model of EAE by interfering with IFN-β-mediated inactivation of encephalitogenic T cells [7]. Therefore, we examined whether fingolimod has beneficial effects in EAE mice given Sema4A-Fc. Although Sema4A-Fc inhibits therapeutic effect of IFN-β, intraperitoneal injection and oral administration of fingolimod reduced the PBL counts and disease severity of EAE mice either with or without Sema4A-Fc administration (Fig 4A and 4C). These data demonstrated therapeutic effects of fingolimod in mice given Sema4A-Fc, consistent with the clinical observation obtained in our present study.

## Discussion

IFN-β and GA have been available as first line therapies for MS for a long time. Moreover, long-term safety has been proven for IFN-β in clinical studies [10]. However, approximately one-third of patients with RRMS respond poorly to IFN-β therapy [11]. Reflecting the clinical demand for establishing more effective DMDs, a number of new drugs such as fingolimod and natalizumab have recently become widely used in clinical settings [12, 13]. Owing to the wide range of newly developed DMDs, new biomarkers that help in selecting the most suitable treatment option are in high demand.

Our previous study showed that Sema4A, one of the class IV semaphorins, was increased in the sera of patients with MS, and those who had high Sema4A levels did not respond to IFN-β therapy. These results indicated that Sema4A could be a potential biomarker for treatment selection in MS. In the current study, we investigated the implications of Sema4A on the efficacy of fingolimod, another DMD for MS.

In our study, fingolimod was proven to be effective in patients with high Sema4A levels. Moreover, fingolimod also improved the severity of EAE mice in the presence of Sema4A-Fc. These results suggested that MS patients with high Sema4A levels can benefit from fingolimod therapy even when they are not responding to IFN-β.

Although the exact mode of action of IFN-β in MS pathogenesis still remains unclear, inhibition of T cell activation and effector Th cell differentiation have been shown to contribute to its effectiveness. In contrast to IFN-β, Sema4A activates T cells and promotes Th1 and Th17 differentiation [6, 14]. Therefore, Sema4A may interfere with the suppressive effects of IFN-β on encephalitogenic T cell generation.

On the other hand, fingolimod inhibits the egress of central memory T cells (TCMs) from lymph nodes. Fingolimod is phosphorylated immediately after oral administration and the phosphorylated form interacts with S1P receptors except for S1P2. Phosphorylated fingolimod inhibits egress of T cells and B cells from lymph nodes, preventing lymphocyte infiltration into the CNS [15].

Since fingolimod inhibits circulating TCMs, including Th17 cells, we hypothesized that fingolimod could have beneficial effects for patients with high Sema4A levels. In support of this hypothesis, fingolimod reduced the PBL counts regardless of the serum Sema4A levels (Figs 2A and 4C) in the present study.

In addition to inhibition of lymphocyte egress from secondary lymphatic organs, fingolimod has been shown to have neuroprotective effects. Fingolimod efficacy was lost in mice with EAE lacking S1P1 on GFAP-expressing astrocytes [16] and therapeutic administration of fingolimod to EAE mice hampered astrocyte activation and nitric oxide production [17]. Sema4A is also expressed on CNS-resident cells, such as microglial cells [18]. To what extent fingolimod has protective effects against Sema4A by modulating the CNS resident neurons and glial cells remains to be elucidated in future studies.

## Conclusions

The current study provides evidence that fingolimod serve as efficacious therapy for MS patients with high Sema4A levels.
